# Transesophageal echocardiography (TEE) in the detection of intraoperative cardiac arrest: A case report

**DOI:** 10.1097/MD.0000000000019928

**Published:** 2020-05-01

**Authors:** Donghang Zhang, Hui Yang, Mingjing Chen, Zihao Zheng, Wenying Zhou, Haibo Song

**Affiliations:** a Department of Anesthesiology, West China Hospital of Sichuan University, Chengdu, Sichuan; b Department of Anesthesiology, Shenzhen People's Hospital, Shenzhen, China.

**Keywords:** cardiac arrest, cardiopulmonary resuscitation, transesophageal echocardiography

## Abstract

**Rationale:**

Point-of-care ultrasound is widely used in patients with cardiac arrest, allowing for diagnosing, monitoring, and prognostication as well as assessing the effectiveness of the chest compressions. However, the detection of intraoperative cardiac arrest by Point-of-care ultrasound was rarely reported.

**Patient concerns:**

A 21-year-old male with Marfan syndrome which manifested Valsalva sinus aneurysms was admitted for aortic valve replacement. After endotracheal intubation, TEE transducer was inserted to evaluate the cardiac structure and function with different views. Severe aortic valve regurgitation was observed in the mid-esophageal aortic valve long and short axis view.

**Diagnosis:**

TEE showed that cardiac contraction was nearly stopped, the spontaneous echo contrast was obvious in the left ventricular and hardly any blood was pumped out from the heart despite the ECG showing normal sinus rhythm with HR 61 beats/min. Meanwhile, the IBP was dropped to 50/30 mm Hg.

**Interventions:**

Chest compressions were started immediately and epinephrine 100 μg was given intravenously. After 30 times of chest compressions, TEE showed that cardiac contractility increased and the stroke volume was improved in the TG SAX view.

**Outcomes:**

The patient was discharged 18 days later in a stable condition.

**Lessons:**

Continuous echocardiography monitoring may be of particular value in forewarning and detecting cardiac arrest in high-risk patients.

## Abstract 2

Supplemental Digital Content is available in the text

## 1 Introduction

Although the incidence of intraoperative cardiac arrest (CA) has dropped with significant advances in physiologic monitoring and surgical anesthetic techniques, it may lead to a severe adverse event with a high mortality once it occurs. Clinical outcomes were not satisfactory in survivors who underwent intraoperative CA.^[1,2]^ Thus, the detection and appropriate interventions of intraoperative CA are very important. One of the main indicators of intraoperative CA is the absence of cardiac rhythm or presence of chaotic cardiac rhythm in the electrocardiogram (ECG).^[3]^ However, there are situations that CA may occur despite the presence of a cardiac rhythm on the monitor, which are typically called pulseless electrical activity (PEA). In these situations, the successful rate of cardiopulmonary resuscitation (CPR) may decrease due to delayed information of the ECG.^[4]^ We reported a case that showed the value of transesophageal echocardiography (TEE) in both the detection of intraoperative CA and the guidance of CPR. The present case report was designed to improve awareness on forewarning and detecting intraoperative CA in high-risk patients with TEE.

## 2 Ethical statement and consent

Informed written consent was obtained from the patient for publication of this case report and accompanying videos.

### 2.1 Case report

A 21-year-old male weighing 65 kg with a history of Marfan syndrome which manifested Valsalva sinus aneurysms was admitted to our hospital for aortic valve replacement. After arriving in the operating room, this patient was monitored with an ECG, and pulse oximetry. The left radial artery was punctured for invasive blood pressure (IBP) monitoring. The initial IBP was 150/60 mm Hg, the ECG showed normal sinus rhythm with heart rate (HR) 130 beats per minute(beats/minute) and oxygen saturation was 98%. General anesthesia was performed after preoxygenation with 6 L/minute oxygen for 3 minutes. Anesthesia was induced with midazolam 3 mg (i.v.), propofol 100 mg (i.v.), rocuronium 40 mg (i.v.), and sufentanil 50 μg (i.v.). After 90 seconds of mask ventilation, oral endotracheal intubation (internal diameter, 7.5 mm) was performed. Then, right internal jugular vein (IJV) catheterization was performed under the guidance of ultrasound and the length of catheterization was about 15 minutes. Transthoracic echocardiography (TTE) (Philips, CX 50, S5-1) was performed during the process from anesthesia induction to IJV catheterization. Severe aortic valve regurgitation was shown in the transthoracic aortic valve short axis view (Supplemental Video 1). Left ventricular hypertrophy, left ventricular wall thinning and wall thickness ratio decrease was shown in the transthoracic left ventricular short axis view (Supplemental Video 2). After the IJV catheterization completed, TEE transducer (Philips, CX 50, X7-2t) was inserted to evaluate the cardiac structure and function with different views. Severe aortic valve regurgitation was observed in the mid-esophageal aortic valve long and short axis view (Supplemental Video 3). A weak cardiac contraction was shown in the transgastric left ventricular short axis (TG SAX) view with a HR of 85 beats/minute (Supplemental Video 5). After 1 minute, the cardiac contraction was nearly stopped, the spontaneous echo contrast was obvious in the left ventricular and hardly any blood was pumped out from the heart despite the ECG showing normal sinus rhythm with HR 61 beats/minute (Supplemental Video 6). Meanwhile, the IBP was dropped to 50/30 mm Hg. Chest compressions were started immediately and epinephrine 100 μg was given intravenously. After 30 times of chest compressions, TEE showed that cardiac contractility increased and the stroke volume was improved in the TG SAX view (Supplemental Video 7). The monitor showed a rise of HR (144 beats/minute) and IBP (160/70 mm Hg). The operation was completed successfully with a duration of 4 hours and the patient was transferred into intensive care unit (ICU) postoperatively. This patient was extubated on the 4th postoperative day. No neurological complications were observed in this patient and he was transferred to general wards on the 6th postoperative day. The patient was discharged from the hospital on the 18th postoperative day.

## 3 Discussion

CA is frequently encountered by physicians in the Emergency Department, ICU, medical/surgical wards, and operating rooms.^[5]^ Point-of-care ultrasound (POCUS) has been proven to be useful in CA in diagnosis, monitoring, and prognosis as well as in assessing the effectiveness of the chest compressions.^[6]^ However, to the best of our knowledge, no previous studies have evaluated the use of POCUS in forewarning and detecting intraoperative CA.

Heart rhythms associated with cardiac arrest include 2 groups: shockable rhythms (ventricular fibrillation/pulseless ventricular tachycardia (VF/pVT)) and non-shockable rhythms (asystole and PEA). Defibrillation can be used for those patients with VF/pVT.^[7]^ POCUS can be used to help differentiate these 2 groups of arrhythmias.^[4]^ ECG monitoring provided limited information about myocardial status during cardiac arrest.^[8]^ Moreover, external artifacts on the ECG can dramatically influence the diagnosis of the underlying heart rhythm. A case of VF masked by ECG was ultimately confirmed by TTE.^[9]^ Therefore, continuous echocardiography monitoring was suggested more appropriate than ECG in CA.

PEA without cardiac contractility was defined as electromechanical dissociation (EMD), and with cardiac contractility as pseudo-EMD.^[10]^ It was reported that the rates of return of spontaneous circulation and the survival upon hospital discharge and after 6 months in patients with pseudo-EMD were higher than the patients with EMD.^[10]^ Studies has shown that 10% to 35% of the CA patients have detectable cardiac contraction with POCUS and have a better prognosis than the patients without cardiac contraction.^[6]^ However, the survivors of CA patients have a higher mortality due to irreversible damage of the brain, myocardium, and other vital organs.^[2]^ In this case, pseudo-EMD was timely detected by TEE, if was not for the rapid treatment, pseudo-EMD might evolve into EMD, and the patient may have severe outcomes.

CA may occur during anesthesia induction, tracheal intubation, and central vena catheterization.^[11]^ This patient was at a high risk for CA during induction, and CA finally happened even though the anesthetists were alerted. Echocardiography monitoring for high-risk patients, especially for the patients with severe aortic valve regurgitation appears to be essential in forewarning and detecting CA during the whole perioperative period. Compared with TTE, TEE is used more widely in CA due to its adequate or sufficient images. And TEE can be used for continuous monitoring without interrupting the chest compressions.^[8]^ However, TTE may be more appropriate to monitor the cardiac status before intubation while TEE is limited by the interference of intubation. The seamless connection between TTE and TEE provided a safer perioperative management.

Recently, Many studies and case reports have demonstrated the successful application of POCUS in Emergency Department during CA.^[8]^ A traumatic case with the aortic valve regurgitation which was not timely diagnosed by TEE could rapidly or progressively lead to congestive heart failure or CA.^[12]^ As for the patients with Valsalva sinus aneurysms and severe aortic valve regurgitation, the successful performance of CPR may be difficult once CA occurs, due to the poor coronary perfusion pressure caused by the increase of left ventricular end-diastolic pressure and the decrease of aortic sinus pressure. Therefore, it is also essential for emergency clinician to evaluate the critically ill patients with the appropriate use of POCUS to prevent CA. For patients in the Emergency Department, TEE is more appropriate than TTE due to the unaffected acoustic window.^[8]^ In this case, TEE showed severe aortic valve regurgitation which was possibly developed into CA due to elevation of left ventricular afterload. Fortunately, we timely detected the occurrence of pseudo-EMD with TEE instead of ECG.

To date, end-tidal CO_2_ and IBP monitoring are still the 2 main indicators recommended in the advanced CPR guidelines.^[7]^ TEE may be a better choice to provide a real-time visualization of the kinetics and size of the cardiac cavities which could be considered as an indicator for continue or terminate resuscitation without interfering chest compressions. It is recommended that critically ill patients should be provided appropriate and frequent monitoring of vital signs, and rapid response to the signs of deterioration and cardiac arrest for prevention of in-hospital cardiac arrest.^[7]^ Echocardiography monitoring may promptly recognize the signs of deterioration and guide interventions to prevent CA. Continuous TTE monitoring with a custom-made TTE transducer holder was reported to be feasible to provide diagnostic information and guide therapeutic management in critically ill patients.^[13]^ Continuous TTE monitoring may also be helpful for transferring the patients who underwent successful CPR. In this patient, TEE represented the most important monitoring tool which provided the signs of CA. Thus, we recommend continuous TEE monitoring as a method for prevention and detection of CA.

Two-thirds of in-hospital cardiac arrests are potentially avoidable. These potentially avoidable in-hospital cardiac arrests may happen due to the delays and errors in diagnosis, inadequate response to signs of deterioration, and incomplete treatment.^[14]^ The significant independent risks of in-hospital cardiac arrests included abnormal breathing indicator, abnormal pulse, and reduced systolic blood pressure.^[15]^ For these patients, continuous echocardiography monitoring may be of particular importance in forewarning and detecting CA.

## Author contributions

Manuscript preparation was by Donghang Zhang and Hui Yang; manuscript review and correction were by Haibo Song and Mingjing Chen; videos were prepared by Zihao Zheng and Wenying Zhou.

**Conceptualization:** Hui Yang, Haibo Song.

**Software:** Zihao Zheng, Wenying Zhou.

**Writing – original draft:** Donghang Zhang, Hui Yang.

**Writing – review & editing:** Mingjing Chen, Haibo Song.

## Supplementary Material

Supplemental Digital Content

## Supplementary Material

Supplemental Digital Content

## Supplementary Material

Supplemental Digital Content

## Supplementary Material

Supplemental Digital Content

## Supplementary Material

Supplemental Digital Content

## Supplementary Material

Supplemental Digital Content

## Supplementary Material

Supplemental Digital Content
